# Cultural validation of the RCADS and use of ensemble learning for symptom profiling of anxiety and depression

**DOI:** 10.3389/fpsyt.2026.1758503

**Published:** 2026-02-27

**Authors:** Zamir Hussain, Mahnoor Hasan, Mehwish Zaman, Syeda Aneela Zahra Shamsi, Qurrat Ulain Hamdan, Haseeba Afzal

**Affiliations:** 1 School of Interdisciplinary Engineering and Sciences (SINES), National University of Sciences and Technology (NUST), Islamabad, Pakistan; 2 Department of Statistical Science, University of Padua, Padova, Italy; 3 Institute of Psychiatry, Rawalpindi Medical University, Rawalpindi, Pakistan

**Keywords:** anxiety, depression, machine learning, mental health, RCADS

## Abstract

**Introduction:**

Depression and anxiety are the most prevalent global mental health concerns, especially among children and adolescents. Numerous screening tools are available to readily detect these issues. The cultural significance of these tools in specific communities should be validated, as socio-demographic factors can influence psychopathology. Moreover, screening tools are limited to the identification of a disorder and do not highlight critical symptoms that may be more dominant in disease progression.

**Methods:**

In this study, a community sample of 237 Pakistani children and adolescents was used to validate the cultural significance of the Revised Child Anxiety and Depression Scale (RCADS) and its subscales, and develop machine learning (ML) models for profiling of the most significant symptoms of anxiety and depression.

**Results:**

Cronbach’s alpha for all subscales of RCADS except Separation Anxiety Disorder (SAD) and Obsessive-Compulsive Disorder (OCD) was above 0.7. Chi-square tests between each item of RCADS and the disorders showed that only gender and grade level of patients did not have statistically significant associations with majority of the scales. Lastly, four ML algorithms were trained where Random Forests exhibited the best performance with accuracies ranging from 0.85 to 0.98. The Gini importance calculated for each item in these models highlights the most dominant symptoms contributing to each disorder.

**Conclusion:**

Overall, the study shows that all 47 individual items in RCADS are culturally significant for the screening of anxiety and depressive disorders in Pakistani populations, however, the subscales for SAD and OCD warrant some modifications due to low Cronbach’s alpha values. The results of ML algorithms yield satisfactory to exceptional metrics, suggesting that these models may be adapted as efficient screening support systems in clinical settings. However, external validation of the models on unseen data is necessary before practical implementation.

## 1 Introduction

Anxiety and depressive disorders are the most prevalent mental disorders, with 301 million and 280 million individuals affected worldwide, respectively (1, 2). Mental disorders can develop at any age, where early years of life like childhood and adolescence are more vulnerable due to being turbulent and dynamic stages of growth and development (3). A meta-analysis published in 2015 estimates the global prevalence at 6.5% and 2.6%, respectively (4). Similar reviews conducted after COVID-19 reveal that the prevalence of childhood anxiety and depression has increased to 20.5% 25.2%, respectively (5). The presentation of these disorders at a young age is often undermined as transient mood lability and emotional bursts during puberty. However, it is estimated that almost 50% of mental disorders diagnosed in adulthood actually begin to develop during childhood and adolescence (6, 7). If these disorders remain untreated, they can become quite debilitating and affect an individual’s quality of life by reducing productivity and socialization. Early detection and timely treatment of these disorders can lead to better outcomes and improved prognosis as compared to later treatment in adulthood (8).

Numerous screening tools are used in clinical practice to readily detect the presence of mental disorders. A few tools used for screening anxiety and depressive disorders in young populations include the Revised Child Anxiety and Depression Scale (RCADS), Screen for Child Anxiety Related Disorders (SCARED), and Spence Children’s Anxiety Scale (SCAS) (9–11). While these tools are convenient to quickly detect the presence or risk of potential issues, they are limited to broadly label patients as “normal”, “borderline”, “at risk”, and/or “clinical”. The specific behaviors or symptoms that predominantly contribute to the development or progression of a disorder cannot be elucidated through these screening measures alone. Such evaluation and symptom profiling is critical for the optimization and personalization of therapeutic strategies to improve patient outcomes (12). However, repeated sessions with patients are required to pinpoint critical behaviors. This process is also dependent on the clinician’s experience and can be subject to clinical bias. An objective and methodical approach is required to highlight dominant symptoms and behaviors early in the screening process to readily formulate personalized treatment plans. The advent of Artificial Intelligence (AI) has introduced computational processes that are able to “learn” from patterns in data and extract critical information based on its mathematical significance or “contribution” in the process. Each variable is assigned a mathematical weight or importance metric depending on its effect upon the final outcome. This technique can be applied on screening tools to identify which specific questions of the tool (and subsequently, the associated symptoms) contribute significantly to disease progression.

Machine Learning (ML) is a field of AI which focuses on the development of computational techniques inspired by the human brain’s ability to learn, adapt, and improve (13). Over the past decade, the predictive capabilities of ML have been extensively researched in the field of healthcare for the development of fast and effective screening tools that can serve as clinical decision supports for healthcare professionals (14, 15). Coupled with clinician’s insights, such ML-based approaches can also prove as beneficial assistants for the prediction of mental disorders (16). Many studies have examined various machine learning algorithms on multidimensional data (demographics, socio-economic information, results of screening and diagnostic tests) for the early detection of depression and anxiety. Haque et al. developed models for the prediction of depression in Australian children using Random Forests with an accuracy of 95% (17), and another research from the same authors reported models based on Gaussian Naïve Bayes and Random Forest to be the best predictors of Obsessive-Compulsive Disorder, Separation Anxiety Disorder, and Attention-Deficit/Hyperactivity Disorder with accuracies ranging between 79-91% (18). A study on Palestinian adolescents used Support Vector Machines to develop models that can predict depression and anxiety at accuracies above 92% (19). Nemesure et al. used biometric and demographic data of university students to develop predictive models using an ensemble approach, where the models provided accuracies of 0.73 for Generalized Anxiety Disorder and 0.67 for Major Depressive Disorder (20). The primary objective of these studies was to develop a quick computational process for the prediction of mental disorders rather than focus on dominant behaviors or patterns for symptom profiling. There are a few studies that have utilized ensemble ML methods for the characterization of social risk factors that affect overall mental well-being of susceptible populations like children exposed to politically volatile circumstances (21). However, the utilization of such techniques for symptom profiling for anxiety and depressive disorders has not been reported thus far.

Moreover, there are no published studies that have analyzed these techniques on a Pakistani cohort. In addition to individual characteristics, environmental and social factors like poverty and inequality can also have detrimental effects on an individual’s mental well-being (3). Such problems are quite prevalent in low-middle income countries (LMICs) like Pakistan, where mental health stigma and uninformed cultural beliefs exacerbate the burden of mental disorders. Mental health conditions are frequently interpreted through moral or religious lenses, often perceived as personal weakness or insufficient faith, which discourages help-seeking behavior and contributes to underdiagnoses (22). The prevalence of collectivist family structures also tends to impose conformity, emotional restraint, and prioritization of family honor over individual psychological needs. Additionally, socioeconomic stressors, political instability, and limited access to mental health services interact with these cultural dynamics, worsening the prevalence and persistence of depression and anxiety in the Pakistani context. Therefore, determining the cultural significance of individual interrogatories or items of screening tools is also required for the optimization of mental healthcare.

The following two aims were central to this study:

i. Develop an ML-based process for the symptom profiling of anxiety and depressive disorders to serve as a foundation for personalized mental healthcare.
ii. Use statistical techniques for the cultural evaluation of the Revised Child Anxiety and Depression Scales using a community sample of Pakistani Children and Adolescents.

## 2 Methods

All statistical and computational processes have been implemented in the programming language Python, version 3.10, using Colab notebooks.

### 2.1 Screening tool

The Revised Child Anxiety and Depression Scale (RCADS) has been selected for this research as it simultaneously screens for different anxiety and depressive disorders in children and adolescents and is widely used by clinicians around the world. It has also been translated in Urdu, the national language of Pakistan, and psychometrically validated in a Pakistani cohort (23). RCADS is a 47-item scale used to screen for the risk of borderline and clinical anxiety and depressive disorders in children and adolescents aged 8 to 18 years (9). All 47 items are used to assess the risk of Total Internalizing Issues. These are then divided into two main scales for Major Depressive Disorder (MDD) and Total Anxiety. The latter scale of anxiety is further divided into 5 subscales for Generalized Anxiety Disorder (GAD), Separation Anxiety Disorder (SAD), Social Phobia or Social Anxiety Disorder (SP), Panic Disorder (PD), and Obsessive Compulsive Disorder (OCD). All these scales are scored using a 4-point system. The initial scores are then converted to corresponding T-scores according to the patient’s gender and grade in school. T-scores below 65 are considered “Normal”, between 65–69 are “Borderline”, and T-scores above 70 might indicate “Clinical” cases, warranting further professional intervention.

### 2.2 Data description

The study received ethical approval from the Institutional Review Board at first author’s affiliated institution under Application No. 2024-IRB-A-06/06 on 22-02-2024. Secondary data collected from both clinical (outpatient psychiatry department) and non-clinical (primary and secondary schools) settings under the supervision of the psychiatrist in our research team was used. For clinical settings, a consecutive sampling method was employed where patients within the age range of 8–18 years referred for emotional problems were recruited. For non-clinical settings, a purposive sampling method was used where teachers referred students who were exhibiting emotional issues. A total of 285 samples were collected, 138 from clinical and 147 from non-clinical settings. As no statistically significant differences between clinical and non-clinical samples were obtained after applying independent sample t-tests (**Table 1**), the dataset was combined.

**Table 1:** Results of independent sample t-tests applied between clinical and non-clinical samples.

No.	Scale/disorder	Levene’s test for equality of variances (p-value)	t-test for equality of means (p-value)
1	Total Internalizing Issues	0.00	0.06
2	Total Anxiety	0.41	0.75
3	MDD	0.00	0.02
4	GAD	0.08	0.37
5	SAD	0.20	0.12
6	SP	0.00	0.08
7	PD	0.00	0.05
8	OCD	0.03	0.24

After pre-processing and removal of missing values (see **Supplementary File: Table 2.docx**), the final dataset consisted of 237 samples with 179 (75.8%) females and 58 (24.6%) males (**Table 2**). While limited, sample sizes in this range have been used in published research, particularly in studies employing behavioral features for classification tasks using machine learning (24–28). The recruited participants were in Primary (25.7%), Middle (32.1%), and High School (42.2%). Their ages ranged from 8 years to 17 years, which have been grouped according to their education level. Scoring and labelling of the participants according to the criteria of RCADS revealed that majority were Normal and not at risk of anxiety and depressive disorders, except for SAD where Normal (42.2%) and Clinical (43.5%) cases were almost the same. Borderline cases were the fewest for all disorders.

**Table 2:** Data description.

No.	Features	Categories	n (%)
1	Gender	Male	58 (24.5)
		Female	179 (75.5)
2	Education	Primary School	61 (25.7)
		Middle School	76 (32.1)
		High School	100 (42.2)
3	Ages	8–10 years	61 (25.7)
		11–13 years	76 (32.1)
		14–17 years	100 (42.2)
4	Prevalence of Internalizing Problems	Normal	157 (66.2)
		Borderline	22 (9.3)
		Clinical	58 (24.5)
5	Prevalence of Overall Anxiety	Normal	132 (55.7)
		Borderline	23 (9.7)
		Clinical	82 (34.6)
6	Prevalence of MDD	Normal	165 (69.6)
		Borderline	21 (8.9)
		Clinical	51 (21.5)
7	Prevalence of GAD	Normal	201 (84.8)
		Borderline	14 (5.9)
		Clinical	22 (9.3)
8	Prevalence of SAD	Normal	100 (42.2)
		Borderline	34 (14.3)
		Clinical	103 (43.5)
9	Prevalence of SP	Normal	147 (62.1)
		Borderline	20 (8.4)
		Clinical	70 (29.5)
10	Prevalence of PD	Normal	204 (86.1)
		Borderline	16 (6.7)
		Clinical	17 (7.2)
11	Prevalence of OCD	Normal	163 (68.8)
		Borderline	31 (13.1)
		Clinical	43 (18.1)

### 2.3 Cultural evaluation of RCADS

#### 2.3.1 Reliability analysis

To evaluate the psychometric properties and reliability of RCADS for Pakistani children and adolescents, a brief reliability analysis is performed on RCADS where Cronbach’s alpha has been estimated for each scale. This is a statistical measure for the reliability and validity of questionnaires or surveys, where values above 0.7 indicate that their constituents correlate adequately, resulting in a satisfactory measure of the problem or factor being evaluated (29). This analysis also describes how the deletion of any items from the questionnaire or survey affects the value of alpha, which informs if any amendments are required to improve overall reliability and validity. Item-total correlations for each constituent are also calculated, where values ranging between 0.2 to 0.6 indicate moderate correlations. This shows that the survey being evaluated focuses on the same topic or theme, but does not contain repetitive or redundant items.

#### 2.3.2 Association analysis

The association of each item of RCADS with the presence of anxiety and depressive disorders has been investigated using Chi-square (χ^2^) tests. The level of significance for these statistical tests has been set at 0.05, where results with a p-value less than 0.05 will be considered statistically significant. This analysis will determine if there are any items in RCADS that are not statistically significant in Pakistani populations. These items will be omitted resulting in a χ^2^-subset of RCADS. The effect of the removal will be evaluated in subsequent steps.

### 2.4 Machine learning for symptom profiling

#### 2.4.1 Resolving class imbalance

From the data description in **Table 2**, it is clear that the three categories of “Normal”, “Borderline”, and “Clinical” are not equally distributed in the data, which leads to a “class imbalance” that can impair the performance of ML algorithms due to bias. This issue has been rectified through Synthetic Minority Over-sampling TEchnique for Nominal data (SMOTE-N). The method generates synthetic minority class instances by randomly selecting a minority instance and identifying its *k* nearest neighbors using the Value Difference Matric (VDM) (30). In this study, default value of *k* = 5 has been applied. Additional synthetic instances were generated for minority classes to match the number of cases of the majority class, ensuring a more balanced distribution in the dataset (**Table 3**).

**Table 3:** Number of instances in the data before and after resampling with SMOTE-N.

No.	Scale/disorder	Categories	Instances before resampling (n)	Instances after resampling (n)
1	Total Internalizing Issues	Normal	157	157
		Borderline	22	157
		Clinical	58	157
2	Total Anxiety	Normal	132	132
		Borderline	23	132
		Clinical	82	132
3	MDD	Normal	165	165
		Borderline	21	165
		Clinical	51	165
4	GAD	Normal	201	201
		Borderline	14	201
		Clinical	22	201
5	SAD	Normal	100	103
		Borderline	34	103
		Clinical	103	103
6	SP	Normal	204	204
		Borderline	16	204
		Clinical	17	204
7	PD	Normal	147	147
		Borderline	20	147
		Clinical	70	147
8	OCD	Normal	163	163
		Borderline	31	163
		Clinical	43	163

#### 2.4.2 ML algorithms

Four ML algorithms viz. Decision Tree (DT), Random Forest (RF), Support Vector Machine (SVM), and Logistic Regression (LR) are selected to develop the proposed predictive models for anxiety and depression. These algorithms have been shortlisted due to their frequent application in related studies (17–19). Primarily, default parameters as defined in the *scikit-learn* library are retained for the ML algorithms after iteratively investigating different hyperparameters until best performance was achieved (31). However, a few modifications in the hyperparameters were made as follows:

- For RF, maximum depth of the trees was set to *10* instead of the default *None*.
- For SVM, the *radial basis function* or *rbf* kernel has been used for multi-class classification.
- For LR, models with *200* iterations have been developed instead of the default of *100*.

The training, testing, and validation of the algorithms has been done using 5-fold cross-validation where data is divided into 5 parts or “folds” that are iteratively used as a testing set. The average performance of the algorithms is then computed by calculating the mean of the accuracies or cross-validation (CV) scores obtained on each testing set. Such techniques limit the possibility of issues like over-fitting, which can impair the algorithm’s performance on real-world data (32).

Average CV scores, accuracy, macro average of recall or sensitivity (number of correct predictions of Borderline and Clinical cases), and macro average of specificity (number of correct predictions of Normal cases) of the algorithms have been used to evaluate model performance. Among these, recall is of utmost importance as the efficiency of clinical decision support is based on its ability to accurately identify Borderline and Clinical cases. These metrics have been analyzed to determine which of the four algorithms results in the most efficient predictive model.

The best algorithm will then be trained separately on the χ^2^-subset of RCADS formed after association analysis. The results obtained using all items and the χ^2^-subset will be compared to determine whether the removal of insignificant items impacts predictive efficiency. In addition to the aforementioned performance metrics, Cohen’s Kappa coefficient (κ) will also be calculated which is a measure of agreement between the ground truth and predictions made by an algorithm. To ensure cohesive results, the algorithms will make predictions on the same test so that obtained values of κ for both sets of features are comparable. Then, McNemar’s test will be performed to determine if the difference in the values of κ is statistically significant. The significance level will be set to 0.05.

From the final algorithm, the significance or importance of predictive features will be determined to highlight the most significant symptoms or factors contributing to specific depressive and anxiety disorders.

## 3 Results

### 3.1 Reliability analysis

**Table 4** shows the Cronbach’s alpha calculated for all scales of RCADS. The value of alpha is above the recommended threshold of 0.7 for all except SAD and OCD, where the values are 0.63 and 0.64, respectively. While these values are below the defined threshold, they are still moderate and can be considered somewhat satisfactory. However, these results reflect that the subscales for SAD and OCD warrant slight modifications for Pakistani pediatric populations. Additionally, deletion of any item from the scales decreases the value of alpha (see **Supplementary File: Table 1.docx**), indicating that the original structure of RCADS constitutes a satisfactory measure for depressive and anxiety disorders in a Pakistani cohort, except for SAD and OCD, where further studies are required, possibly in regards to the addition of more items or the modification of present items.

**Table 4:** Values of Cronbach’s alpha for all scales of RCADS.

No	Scale/disorder	Cronbach’s alpha
1	Total Internalizing Issues	0.93
2	Total Anxiety	0.91
3	MDD	0.83
4	GAD	0.72
5	SAD	0.63
6	SP	0.78
7	PD	0.81
8	OCD	0.64

### 3.2 Association analysis

Majority of the items showed significant association with respective disorders, which corroborates with the satisfactory alpha values obtained during the reliability analysis. Only a few variables did not have significant results which are shown in **Table 5**. For all scales, the variable of Gender is not significantly associated. Grade is not significantly associated with Total Anxiety, MDD, GAD, SAD, SP, and PD, while Total Internalizing Issues is the only scale where 2 items of RCADS are not significantly associated. These variables have been removed to create χ^2^-subsets of each scale, which have been subsequently used to train ML algorithms to determine whether their removal influences predictive performance.

**Table 5:** Items of RCADS that do not have statistically significant associations with the disorders.

No.	Scale/disorder	Items	χ^2^	P-value
1	Total Internalizing Issues	Item 5: I would feel afraid of being on my own at home	5.16	0.52
		Item 9: I worry about being away from my parents	5.15	0.52
		Gender	1.23	0.54
2	Total Anxiety	Gender	0.52	0.77
		Grade	27.29	0.13
3	MDD	Gender	1.07	0.59
		Grade	26.29	0.16
4	GAD	Gender	1.88	0.39
		Grade	22.11	0.34
5	SAD	Gender	0.16	0.92
		Grade	26.68	0.14
6	SP	Gender	3.37	0.18
		Grade	24.07	0.24
7	PD	Gender	3.46	0.18
		Grade	23.19	0.21
8	OCD	Gender	4.02	0.13

### 3.3 Machine learning for symptom profiling

The performance of the four algorithms is compared with each other to deduce the most efficient predictive model (**Table 6**). For all scales, RF results in the highest metrics. SVM provides the second best results, with the performance being quite comparable for the scales of Total Internalizing Issues, SAD, and SP, where both algorithms exhibit the same specificity.

**Table 6:** Performance metrics of the four algorithms trained on all features for all scales of RCADS.

No.	Scale/disorder	Algorithm	Performance metrics			
			Accuracy	Recall	Specificity	Average CVS
1	Total Internalizing Issues	*DT*	0.88	0.88	0.94	0.88
		*RF*	0.95	0.95	0.97	0.95
		*SVM*	0.94	0.94	0.97	0.94
		*LR*	0.93	0.93	0.96	0.93
2	Total Anxiety	*DT*	0.84	0.84	0.91	0.84
		*RF*	0.90	0.90	0.95	0.90
		*SVM*	0.88	0.88	0.94	0.88
		*LR*	0.87	0.87	0.93	0.87
3	MDD	*DT*	0.90	0.90	0.95	0.90
		*RF*	0.96	0.96	0.98	0.96
		*SVM*	0.93	0.93	0.97	0.93
		*LR*	0.93	0.93	0.96	0.93
4	GAD	*DT*	0.96	0.96	0.97	0.95
		*RF*	0.97	0.97	0.98	0.97
		*SVM*	0.92	0.92	0.96	0.92
		*LR*	0.90	0.90	0.95	0.90
5	SAD	*DT*	0.78	0.78	0.88	0.79
		*RF*	0.85	0.85	0.91	0.84
		*SVM*	0.84	0.84	0.91	0.84
		*LR*	0.83	0.83	0.91	0.83
6	SP	*DT*	0.96	0.96	0.98	0.96
		*RF*	0.98	0.98	0.99	0.98
		*SVM*	0.97	0.97	0.99	0.97
		*LR*	0.97	0.97	0.98	0.97
7	PD	*DT*	0.89	0.89	0.94	0.89
		*RF*	0.92	0.92	0.96	0.93
		*SVM*	0.88	0.88	0.94	0.88
		*LR*	0.88	0.88	0.93	0.88
8	OCD	*DT*	0.88	0.88	0.93	0.87
		*RF*	0.93	0.93	0.97	0.93
		*SVM*	0.89	0.89	0.94	0.89
		*LR*	0.87	0.87	0.93	0.87

**Figure 1:**
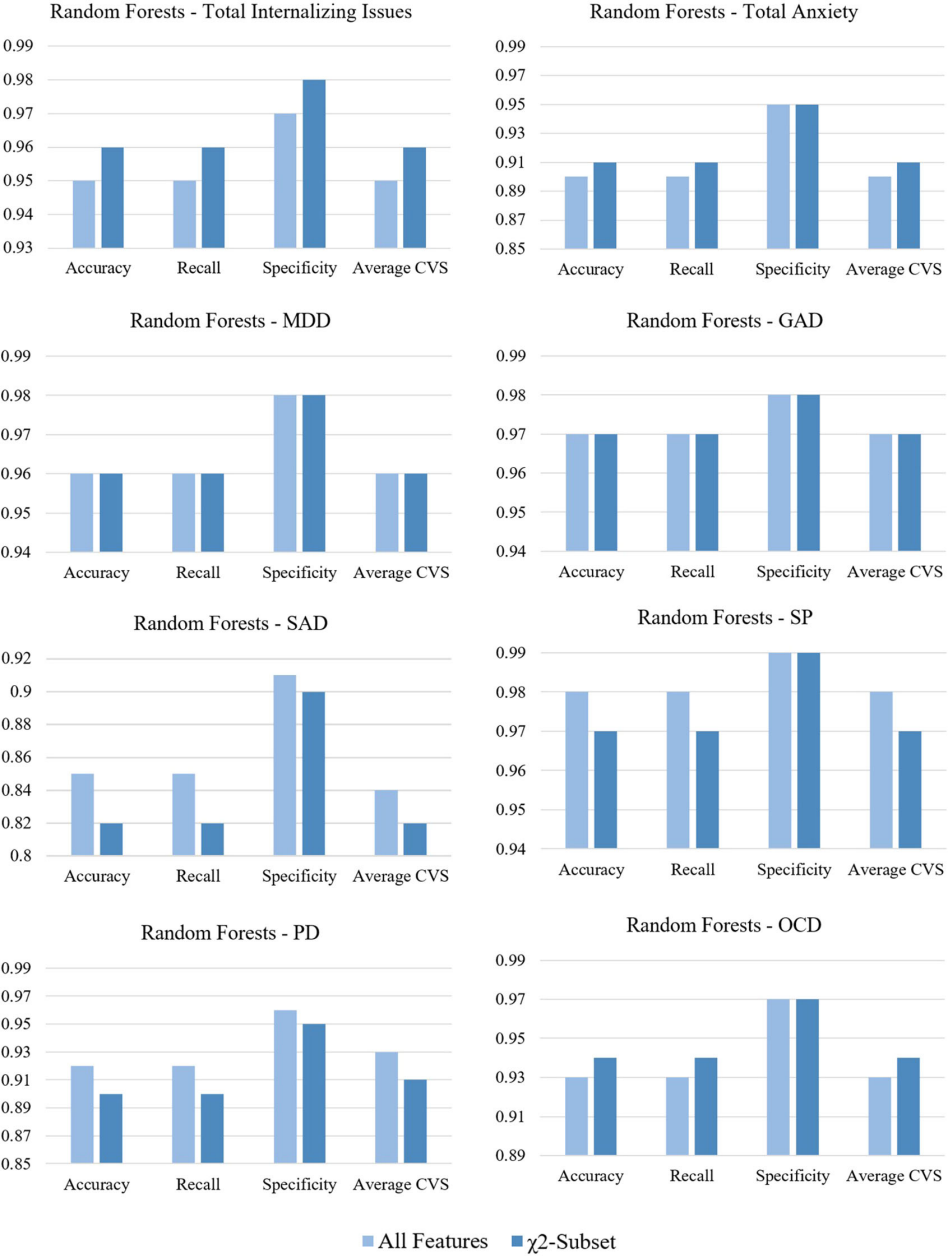
Comparison between random forests trained on all features of RCADS and the χ^2^-subsets.

In the next step, the performance of RF trained using all items of RCADS and the χ^2^-subsets have been compared (**Figure 1**). In case of Total Internalizing Issues, all metrics improve after removal of Item 5, Item 9, and Gender. In case of Total Anxiety accuracy and recall improve after the removal of Grade and Gender, and in case of OCD, the same improvement is observed after removal of Gender only. For MDD and GAD, the metrics remain unchanged, however, the confusion matrices in **Table 7** reveal that RFs trained on the χ^2^-subsets are slightly better at making true predictions. In case of SAD, SP, and PD, the performance of RF decreases after the removal of Grade and Gender.

**Table 7:** Confusion matrices of random forests trained on all features and the χ^2^-subsets for MDD and GAD.

No.	Disorder	Set of features	Class labels		Predicted		
					Normal	Borderline	Clinical
1	MDD	All features	Actual	Normal	160	3	2
				Borderline	5	154	6
				Clinical	4	1	160
		χ^2^-subset		Normal	161	2	2
				Borderline	6	154	5
				Clinical	5	0	160
2	GAD	All features		Normal	195	3	3
				Borderline	5	194	2
				Clinical	8	1	192
		χ^2^-subset		Normal	196	1	4
				Borderline	2	196	3
				Clinical	6	2	193

From the previous comparison of performance metrics, a marginal difference is observed between the algorithms trained on all features and the χ^2^-subset. Further evaluation was required for more conclusive results. Therefore, Cohen’s Kappa coefficient (κ) was also evaluated by using the RF algorithms to make predictions on the same test sets (**Table 8**). Comparison between the values of κ also yield a similar trend of results as before. In case of Total Internalizing Issues, algorithms trained on the χ^2^-subset have higher values of κ showing that this subset resulted in better model agreement between ground truth and predictions. In case of Total Anxiety, MDD, GAD, and OCD, no difference is observed in the values of κ. For SAD, SP, and PD, the value of κ is higher for models trained on all features. For the scales where differences in the values of κ are observed, McNemar’s test was also applied, where all tests had p-values above 0.05. This confirms that any difference in the performance of the algorithms has occurred by chance.

**Table 8:** Cohen’s Kappa coefficient for random forests trained on all features and the χ^2^-subset.

No	Scale/disorder	Cohen’s kappa coefficient (κ)		McNemar’s test (p-value)
		All features	χ^2^-subset	
1	Total Internalizing Issues	0.92	0.94	0.81
2	Total Anxiety	0.87	0.87	–
3	MDD	0.94	0.94	–
4	GAD	0.95	0.95	–
5	SAD	0.77	0.73	0.09
6	SP	0.96	0.95	0.72
7	PD	0.89	0.86	0.93
8	OCD	0.91	0.91	–

For symptom profiling, the Gini importance of the features used to train the final RF models was analyzed. This is a measure for the reduction in misclassification or Gini impurity contributed by each feature in the model. Higher values of Gini importance indicate higher contribution of a feature towards the reduction of impurity or error. For each scale of RCADS, the top 3 features with the highest Gini importance are discussed ahead (**Table 9**). As Gini importance can be biased and inflate the significance of features (33), a permutation test was also applied at a significance level of 0.05. The p-values of the permutation are also reported which show that the observed importance of the features in statistically significant.

**Table 9:** Top 3 features with the highest Gini importance for each random forest of RCADS and its subscales.

No.	Scale/disorder	Top 3 features	Gini importance	Permutation test (p-value)
1	Total Internalizing Issues	Item 34: All of a sudden I feel really scared for no reason at all	0.08	0.00
		Item 15: I have problems with my appetite	0.07	0.00
		Item 41: I worry that I will suddenly get a scared feeling when there is nothing to be afraid of	0.06	0.02
2	Total Anxiety	Item 24: When I have a problem, my heart beats really fast	0.08	0.01
		Item 4: I worry when I think I have done poorly at something	0.08	0.00
		Item 27: I worry that something bad will happen to me	0.06	0.00
3	MDD	Item 40: I feel like I don’t want to move	0.23	0.01
		Item 6: Nothing is much fun anymore	0.16	0.01
		Item 19: I have no energy for things	0.14	0.01
4	GAD	Item 27: I worry that something bad will happen to me	0.31	0.02
		Item 22: I worry that bad things will happen to me	0.19	0.01
		Item 13: I worry that something awful will happen to someone in my family	0.15	0.01
5	SAD	Item 46: I would feel scared if I had to stay away from home overnight	0.18	0.02
		Item 18: I have trouble going to school in the mornings because I feel nervous or afraid	0.15	0.01
		Item 17: I feel scared if I have to sleep on my own	0.15	0.02
6	SP	Item 32: I worry what other people think of me	0.16	0.04
		Item 38: I feel afraid if I have to talk in front of my class	0.15	0.01
		Item 20: I worry I might look foolish	0.14	0.00
7	PD	Item 34: All of a sudden I feel really scared for no reason at all	0.22	0.01
		Item 26: I suddenly start to tremble or shake when there is no reason for this	0.15	0.00
		Item 39: My heart suddenly starts to beat too quickly for no reason	0.14	0.01
8	OCD	Item 23: I can’t seem to get bad or silly thoughts out of my head	0.22	0.01
		Item 31: I have to think of special thoughts (like numbers or words) to stop bad things from happening	0.19	0.01
		Item 10: I get bothered by bad or silly thoughts or pictures in my mind	0.16	0.01

## 4 Discussion

The top 3 features elucidated by the models show that in case of Total Internalizing Issues, two items pertaining to unexplainable anxiety and one item regarding issues with appetite are the most dominant. For Total Anxiety, accelerated heartbeat due to anxiety, being worried about poor performance, and a general fear about something bad happening to oneself are the top 3 symptoms. For MDD, the top 3 features relate to the depressive symptoms of lethargy and lack of interest. In case of GAD, general fears regarding bad things happening to oneself or a loved one dominate, and for SAD, being away from home, avoiding or being anxious about going to school, and sleeping alone appear on top. For SP, fears related to how perceive oneself, presenting in front of an audience, and appearing foolish are the top 3 symptoms. In case of PD, the three features are related to unexplainable increase in fear, trembling, and faster heartrate. For OCD, obsessive thoughts are the dominant features as opposed to compulsive acts. For each individual, RF models can highlight a list of the most significant features or symptoms, which can be used to formulate personalized therapies for each individual, where the most critical behaviors are addressed expeditiously.

Similar studies on the predictive modelling or classification of anxiety and depressive disorders either utilize tree-based ensemble ML algorithms like RF and XGBoost, or report them as the best performing model from a pool of different classical and ensemble algorithms (17, 18, 20, 34). Similar results were also obtained in the present research, where RF outperformed other algorithms. This is attributed to the ensemble nature of the algorithm which optimizes output by the aggregation of multiple decision trees, and its inherent ability to deal with potential non-linear relationships among features (35). This may also be attributed to the nature of the data and classification task, which is fairly straightforward as the numerical value of a few features elucidate how each instance will be classified in to the three classes of Normal, Borderline, and Clinical. However, it is worth mentioning that the performance of SVM is comparable to RF in some cases. Qasrawi et al. also report better performance of SVM for the classification of depression and anxiety in their research (19). SVM is also a powerful classification algorithm that performs well on the type of dataset used in this research. However, the reason for preference of RF over SVM in this regard is not merely informed by the slightly better performance of RF and the recurrence of the algorithm in quoted literature. Random Forests are more robust and scalable algorithms which can handle non-linear data without the requirement of pre-standardization and deal with missing information via imputation (35). Support Vector Machines require complete and scaled data in order to perform efficiently (36). As the wholeness of medical data is not guaranteed in real-world application, Random Forests might prove to be a more practical choice as an efficient decision support system for the screening of mental disorders.

In the final models selected for each scale of RCADS, Gender has been removed as a predictive feature for Total Internalizing Issues, Total Anxiety, MDD, GAD, and OCD based on the performance metrics. This is interesting as the original T-scoring of RCADS is dependent on the gender of the patient, and research also explains a distinction between psychopathology of males and females (37). In contrast, removing the attribute of gender from the models for SAD, SP, and PD reduces their performance. Similar results are obtained for the attribute of grade level, where its removal from the models of Total Anxiety, MDD, and GAD improves model performance, while the metrics of models for SAD, SP, and PD reduce. Additionally, in case of Total Internalizing Issues, the removal of Items 5 and 9 improve model performance. These items of RCADS pertain to the specific behaviors associated with SAD, therefore, they may not be considered the primary contributors or predictors of general internalizing behavior. However, as these items are retained in the specific model for SAD, their omission from the scale of Total Internalizing Issues seems counter-intuitive. For a more robust comparison, the final RF models were further evaluated on the same test set, and Cohen’s kappa was calculated for models trained on all features and the χ^2^-subsets. This additional testing also revealed contrasting results, where values of κ either increased, decreased, or remained unchanged for some scales. For scales, where any difference in the value of κ was observed, McNemar’s test confirmed that the differences were not statistically significant. Due to the conflicting nature of the outcomes and the proof that any difference in model performance has occurred by chance, it is concluded that no changes need to be made in the composition of RCADS and its interrogatories based on this portion of the research alone. All items within RCADS are culturally significant in a Pakistani cohort, which is to be expected from a widely-used and validated psychometric scale curated by experienced professionals. However, the lower Cronbach’s alpha values obtained for the scales of SAD and OCD indicate that further studies regarding the composition of these subscales with respect to Pakistani populations are required.

It is important to discern that the aim of AI-driven decision support systems is not to provide a definitive diagnosis and, ultimately, a “replacement” for traditional clinical practices. The role of these proposed tools is the enhancement of the existing clinical process. In resource-limited areas like Pakistan, psychiatrists are greatly outnumbered by potential patients. Reportedly, there is only one fully trained mental health professional for every 360,000 patients in Pakistan (38). The statistics become even more drastic for younger patients, with only 1% of the outpatient mental health facilities specializing in child and adolescent psychiatry (22). The implementation of an additional screening aid in the clinical workflow could alleviate the burden of mental health professionals. The speed of AI-driven tools enables them to screen multiple individuals simultaneously, and their efficiency can allow the prioritization of patients that require urgent review from a professional. As the influx of patients in child and adolescent psychiatry departments is quite low, it would be more beneficial to equip general or pediatric outpatient departments with these decision support systems. In addition to conventional check-ups, if parents or frontline healthcare providers are concerned about the mental well-being of their child or patient, they can quickly screen them using the proposed predictive models. This preliminary screening can then inform if the patient should be referred to a mental health specialist, and the symptom profiling can facilitate the specialist in forming patient-centered therapeutic regimes.

While the study establishes the importance of each item of RCADS and provides RF algorithms that can screen mental disorders efficiently, there are certain limitations that may affect the generalizability of the proposed models. Firstly, the sample size is quite small. Although similar sample sizes have been reported in related literature (24–28), ML algorithms require extensive data for optimal performance. While the implementation of SMOTE-N slightly increases the dataset, this is an artificial or synthetic inflation of data, which is not comparable to real instances that are collected organically. Secondly, the gender distribution is quite imbalanced which can be attributed to the sampling methods used for data collection. Both consecutive and purposive sampling are non-probability sampling techniques which can induce bias. However, these methods were employed for convenience, as data collection for mental health research poses some hurdles due to limited clinical cases and socio-cultural implications. Additionally, the reported predisposition of affective disorders in females may also contribute to the gender imbalance (37). Thirdly, the data features only consist of gender, grade, and the 47 items of RCADS. Socio-cultural attributes like family structure, socioeconomic status, and parental education levels are primary determinants of a child’s cognitive development (39, 40). Therefore, their inclusion with questions regarding behaviors and personality traits will result in inclusive and comprehensive screening tools.

In terms of future prospects of this study, the proposed models should be further optimized using a larger and uniform dataset. Socio-demographic and biometric indicators of patients should also be incorporated for more robust cultural adaptation of proposed decision support systems. This study serves as a foundation for the predictive modelling and symptom profiling of depressive and anxiety disorders in at-risk children and adolescents using ubiquitous and standard screening practices. It shows that simple computational approaches can provide efficient decision support systems for healthcare professionals to alleviate their workload and optimize patient outcomes. However, it is appreciated that the implementation of such technological interventions requires extensive validation on external datasets that the models have not been exposed to during initial training. This external validation will investigate the generalizability and reproducibility of the models on varying cohorts.

## Data Availability

The raw data supporting the conclusions of this article will be made available by the authors, without undue reservation.
