# Supplementary material for: Cultural validation of the RCADS and use of ensemble learning for symptom profiling of anxiety and depression
Source: Front Psychiatry. 2026 Feb 27;17:1758503. doi: 10.3389/fpsyt.2026.1758503 (PMC12982416; doi:10.3389/fpsyt.2026.1758503)
Supplement: Supplementary file 1 [file Table1.docx]

Table S1: Corrected Item-Total Correlations and Effect of Deletion of any items on Cronbach's alpha for the Total Internalizing Issues scale of RCADS.

| Item No. in RCADS | Statements | Corrected Item-Total Correlation | Cronbach’s alpha if item deleted |
| --- | --- | --- | --- |
| 1 | I worry about things | 0.39 | 0.93 |
| 2 | I feel sad or empty | 0.48 | 0.93 |
| 3 | I get a funny feeling in stomach | 0.22 | 0.93 |
| 4 | I worry when I think I have done something poorly | 0.35 | 0.93 |
| 5 | I feel afraid of being alone at home | 0.27 | 0.93 |
| 6 | Nothing is much fun anymore | 0.39 | 0.93 |
| 7 | I feel scared when taking a test | 0.32 | 0.93 |
| 8 | I worry when I think someone is angry with me | 0.33 | 0.93 |
| 9 | I worry about being away from my parent | 0.25 | 0.93 |
| 10 | I am bothered by bad/silly thoughts or pictures in my mind | 0.50 | 0.93 |
| 11 | I have trouble sleeping | 0.41 | 0.93 |
| 12 | I worry that I will do badly at school work | 0.58 | 0.93 |
| 13 | I worry that something awful will happen to someone in my family | 0.42 | 0.93 |
| 14 | I suddenly feel that I can’t breathe without any reason | 0.53 | 0.93 |
| 15 | I have problems with my appetite | 0.49 | 0.93 |
| 16 | I have to keep checking that I have done things right | 0.28 | 0.93 |
| 17 | I feel scared if I have to sleep on my own | 0.31 | 0.93 |
| 18 | I have trouble going to school because I feel nervous | 0.38 | 0.93 |
| 19 | I have no energy for things | 0.54 | 0.93 |
| 20 | I worry I might look foolish | 0.56 | 0.93 |
| 21 | I am tired a lot | 0.63 | 0.93 |
| 22 | I worry that bad things will happen to me | 0.67 | 0.93 |
| 23 | I can't seem to get bad/silly thoughts out of my head | 0.54 | 0.93 |
| 24 | My heart beats very fast when I have a problem | 0.44 | 0.93 |
| 25 | I can't think clearly | 0.42 | 0.93 |
| 26 | I suddenly start to tremble or shake when there is no reason | 0.54 | 0.93 |
| 27 | I worry that something bad will happen to me | 0.72 | 0.93 |
| 28 | I feel shaky when I have a problem | 0.52 | 0.93 |
| 29 | I feel worthless | 0.62 | 0.93 |
| 30 | I worry about making mistakes | 0.54 | 0.93 |
| 31 | I have to think of special thoughts to stop bad things from happening | 0.44 | 0.93 |
| 32 | I worry what other people think of me | 0.58 | 0.93 |
| 33 | I am afraid of being in crowded places | 0.39 | 0.93 |
| 34 | I feel really scared suddenly for no reason | 0.64 | 0.93 |
| 35 | I worry about what is going to happen | 0.45 | 0.93 |
| 36 | I suddenly become dizzy or faint when there is no reason | 0.42 | 0.93 |
| 37 | I think about death | 0.49 | 0.93 |
| 38 | I feel afraid if I have to talk in front of class | 0.51 | 0.93 |
| 39 | My heart suddenly starts to beat too quickly for no reason | 0.56 | 0.93 |
| 40 | I feel like I don't want to move | 0.50 | 0.93 |
| 41 | I worry that I will suddenly get a bad feeling when there is nothing to be afraid of | 0.62 | 0.93 |
| 42 | I have to do some things over and over again | 0.28 | 0.93 |
| 43 | I feel afraid that I will make a fool of myself in front of people | 0.50 | 0.93 |
| 44 | I have to do some things in just the right way to stop bad things | 0.35 | 0.93 |
| 45 | I worry when I go to bed at night | 0.62 | 0.93 |
| 46 | I would feel scared if I had to stay away from home overnight | 0.32 | 0.93 |
| 47 | I feel restless | 0.61 | 0.93 |

Table S2: Corrected Item-Total Correlations and Effect of Deletion of any items on Cronbach's alpha for the Total Anxiety scale of RCADS.

| Item No. in RCADS | Statements | Corrected Item-Total Correlation | Cronbach’s alpha if item deleted |
| --- | --- | --- | --- |
| 1 | I worry about things | 0.38 | 0.91 |
| 3 | I get a funny feeling in stomach | 0.20 | 0.91 |
| 4 | I worry when I think I have done something poorly | 0.38 | 0.91 |
| 5 | I feel afraid of being alone at home | 0.30 | 0.91 |
| 7 | I feel scared when taking a test | 0.33 | 0.91 |
| 8 | I worry when I think someone is angry with me | 0.34 | 0.91 |
| 9 | I worry about being away from my parent | 0.28 | 0.91 |
| 10 | I am bothered by bad/silly thoughts or pictures in my mind | 0.48 | 0.91 |
| 12 | I worry that I will do badly at school work | 0.57 | 0.91 |
| 13 | I worry that something awful will happen to someone in my family | 0.46 | 0.91 |
| 14 | I suddenly feel that I can’t breathe without any reason | 0.51 | 0.91 |
| 16 | I have to keep checking that I have done things right | 0.31 | 0.91 |
| 17 | I feel scared if I have to sleep on my own | 0.32 | 0.91 |
| 18 | I have trouble going to school because I feel nervous | 0.37 | 0.91 |
| 20 | I worry I might look foolish | 0.53 | 0.91 |
| 22 | I worry that bad things will happen to me | 0.68 | 0.91 |
| 23 | I can't seem to get bad/silly thoughts out of my head | 0.52 | 0.91 |
| 24 | My heart beats very fast when I have a problem | 0.46 | 0.91 |
| 26 | I suddenly start to tremble or shake when there is no reason | 0.49 | 0.91 |
| 27 | I worry that something bad will happen to me | 0.71 | 0.91 |
| 28 | I feel shaky when I have a problem | 0.50 | 0.91 |
| 30 | I worry about making mistakes | 0.55 | 0.91 |
| 31 | I have to think of special thoughts to stop bad things from happening | 0.47 | 0.91 |
| 32 | I worry what other people think of me | 0.59 | 0.91 |
| 33 | I am afraid of being in crowded places | 0.40 | 0.91 |
| 34 | I feel really scared suddenly for no reason | 0.62 | 0.91 |
| 35 | I worry about what is going to happen | 0.47 | 0.91 |
| 36 | I suddenly become dizzy or faint when there is no reason | 0.38 | 0.91 |
| 37 | I think about death | 0.45 | 0.91 |
| 38 | I feel afraid if I have to talk in front of class | 0.49 | 0.91 |
| 39 | My heart suddenly starts to beat too quickly for no reason | 0.55 | 0.91 |
| 41 | I worry that I will suddenly get a bad feeling when there is nothing to be afraid of | 0.60 | 0.91 |
| 42 | I have to do some things over and over again | 0.28 | 0.91 |
| 43 | I feel afraid that I will make a fool of myself in front of people | 0.49 | 0.91 |
| 44 | I have to do some things in just the right way to stop bad things | 0.36 | 0.91 |
| 45 | I worry when I go to bed at night | 0.58 | 0.91 |
| 46 | I would feel scared if I had to stay away from home overnight | 0.35 | 0.91 |

Table S3: Corrected Item-Total Correlations and Effect of Deletion of any items on Cronbach's alpha for the MDD subscale of RCADS.

| Item No. in RCADS | Items | Corrected Item-Total Correlation | Cronbach’s alpha if item deleted |
| --- | --- | --- | --- |
| 2 | I feel sad or empty | 0.53 | 0.81 |
| 6 | Nothing is much fun anymore | 0.46 | 0.82 |
| 11 | I have trouble sleeping | 0.37 | 0.83 |
| 15 | I have problems with my appetite | 0.48 | 0.82 |
| 19 | I have no energy for things | 0.58 | 0.80 |
| 21 | I am tired a lot | 0.58 | 0.80 |
| 25 | I cannot think clearly | 0.39 | 0.82 |
| 29 | I feel worthless | 0.66 | 0.80 |
| 40 | I feel like I don’t want to move | 0.50 | 0.81 |
| 47 | I feel restless | 0.57 | 0.80 |

Table S4: Corrected Item-Total Correlations and Effect of Deletion of any items on Cronbach's alpha for the GAD subscale of RCADS.

| Item No. in RCADS | Statements | Corrected Item-Total Correlation | Cronbach’s alpha if item deleted |
| --- | --- | --- | --- |
| 1 | I worry about things | 0.32 | 0.72 |
| 13 | I worry that something awful will happen to someone in my family | 0.35 | 0.72 |
| 22 | I worry that bad things will happen to me | 0.62 | 0.63 |
| 27 | I worry that something bad will happen to me | 0.67 | 0.62 |
| 35 | I worry about what is going to happen | 0.41 | 0.70 |
| 37 | I think about death | 0.40 | 0.70 |

Table S5: Corrected Item-Total Correlations and Effect of Deletion of any items on Cronbach's alpha for the SAD subscale of RCADS.

| Item No. in RCADS | Statements | Corrected Item-Total Correlation | Cronbach’s alpha if item deleted |
| --- | --- | --- | --- |
| 5 | I feel afraid of being alone at home | 0.34 | 0.59 |
| 9 | I worry about being away from my parent | 0.44 | 0.56 |
| 17 | I feel scared if I have to sleep on my own | 0.41 | 0.57 |
| 18 | I have trouble going to school because I feel nervous | 0.22 | 0.56 |
| 33 | I am afraid of being in crowded places | 0.35 | 0.59 |
| 45 | I worry when I go to bed at night | 0.32 | 0.59 |
| 46 | I would feel scared if I had to stay away from home overnight | 0.40 | 0.57 |

Table S6: Corrected Item-Total Correlations and Effect of Deletion of any items on Cronbach's alpha for the SP sub scale of RCADS.

| Item No. in RCADS | Statements | Corrected Item-Total Correlation | Cronbach’s alpha if item deleted |
| --- | --- | --- | --- |
| 4 | I worry when I think I have done something poorly | 0.36 | 0.77 |
| 7 | I feel scared when taking a test | 0.32 | 0.79 |
| 8 | I worry when I think someone is angry with me | 0.28 | 0.78 |
| 12 | I worry that I will do badly at school work | 0.54 | 0.75 |
| 20 | I worry I might look foolish | 0.55 | 0.74 |
| 30 | I worry about making mistakes | 0.57 | 0.74 |
| 32 | I worry what other people think of me | 0.60 | 0.74 |
| 38 | I feel afraid if I have to talk in front of class | 0.46 | 0.76 |
| 43 | I feel afraid that I will make a fool of myself in front of people | 0.52 | 0.75 |

Table S7: Corrected Item-Total Correlations and Effect of Deletion of any items on Cronbach's alpha for PD subscale of RCADS.

| Item No. in RCADS | Statements | Corrected Item-Total Correlation | Cronbach’s alpha if item deleted |
| --- | --- | --- | --- |
| 3 | I get a funny feeling in stomach | 0.26 | 0.81 |
| 14 | I suddenly feel that I can’t breathe without any reason | 0.57 | 0.79 |
| 24 | My heart beats very fast when I have a problem | 0.42 | 0.81 |
| 26 | I suddenly start to tremble or shake when there is no reason | 0.59 | 0.78 |
| 28 | I feel shaky when I have a problem | 0.54 | 0.79 |
| 34 | I feel really scared suddenly for no reason | 0.63 | 0.78 |
| 36 | I suddenly become dizzy or faint when there is no reason | 0.48 | 0.80 |
| 39 | My heart suddenly starts to beat too quickly for no reason | 0.58 | 0.79 |
| 41 | I worry that I will suddenly get a bad feeling when there is nothing to be afraid of | 0.56 | 0.79 |

Table S8: Corrected Item-Total Correlations and Effect of Deletion of any items on Cronbach's alpha for the OCD subscale of RCADS.

| Item No. in RCADS | Statements | Corrected Item-Total Correlation | Cronbach’s alpha if item deleted |
| --- | --- | --- | --- |
| 10 | I am bothered by bad/silly thoughts or pictures in my mind | 0.30 | 0.62 |
| 16 | I have to keep checking that I have done things right | 0.33 | 0.61 |
| 23 | I can't seem to get bad/silly thoughts out of my head | 0.44 | 0.57 |
| 31 | I have to think of special thoughts to stop bad things from happening | 0.42 | 0.58 |
| 42 | I have to do some things over and over again | 0.34 | 0.61 |
| 44 | I have to do some things in just the right way to stop bad things | 0.39 | 0.59 |
