# Supplementary material for: Cultural validation of the RCADS and use of ensemble learning for symptom profiling of anxiety and depression
Source: Front Psychiatry. 2026 Feb 27;17:1758503. doi: 10.3389/fpsyt.2026.1758503 (PMC12982416; doi:10.3389/fpsyt.2026.1758503)
Supplement: Supplementary file 2 [file Table2.docx]

Only the clinical data had missing information for 9 items of RCADS. The missing rates of these 9 items are given in Table S1 below.

Table S1: Missing rates of RCADS items in the clinical data.

| No. | Item | No. of missing instances | Missing Rate |
| --- | --- | --- | --- |
| 1 | 6 | 1 | 0.7% |
| 2 | 10 | 1 | 0.7% |
| 3 | 17 | 1 | 0.7% |
| 4 | 18 | 2 | 1.4% |
| 5 | 24 | 1 | 0.7% |
| 6 | 33 | 1 | 0.7% |
| 7 | 35 | 22 | 15.9% |
| 8 | 39 | 1 | 0.7% |
| 9 | 41 | 1 | 0.7% |

A total of 25 instances had one or more missing RCADS items. 48 instances were also missing information about the grade and/or gender of the participants. There were 9 instances with missing information for both grade and gender, 3 instances with missing information for gender only, and 36 instances with missing information for grade only. As RCADS T-scores depend on grade and gender, these 48 instances could not be scored. Therefore, they were removed from the data. Coincidentally, the 25 instances with missing RCADS items were a subset of the 48 instances with missing grade and/or gender information (Figure S1). Hence, removal of the 48 instances also removed instances with missing RCADS items.

Figure S1: Venn diagram showing how the instances with missing grade and/or gender and missing RCADS items interact.

48 instances with missing grade and/or gender

25 instances with missing RCADS items
